# Management of obstructive sleep apnea-hypopnea syndrome in children: what is the role of orthodontics? A scoping review

**DOI:** 10.1007/s11325-025-03288-1

**Published:** 2025-03-13

**Authors:** Margaux Picherit, Thomas Trentesaux, Amandine Ternisien, Nathalie Foumou, Caroline Delfosse, Thomas Marquillier

**Affiliations:** 1 Pratique libérale pédiatrique exclusive, Rouen, F-76000 Rouen France; 2 Univ. Lille, CHU Lille, Odontologie pédiatrique, Lille, F-59000 France; 3 Pratique libérale pédiatrique exclusive, Hallennes-lez-Haubourdin, F-62220 France; 4 Univ. Lille, CHU Lille, Orthopédie dento-faciale, Lille, F-59000 France; 5 Pratique libérale ODF, Carvin, F-62220 France; 6 Laboratoire Éducations et Promotion de la Santé, LEPS UR3412, Université Sorbonne Paris Nord, Bobigny, 93017 France

**Keywords:** Respiration disorders, Sleep wake disorders, Sleep apnoea syndromes, Sleep apnoea, Obstructive

## Abstract

**Purpose:**

Obstructive sleep apnoea syndrome (OSAHS) is a respiratory disorder that greatly affects the health and quality of life of patients. OSAHS affects up to 5.7% of children aged up to 18 years old, and its prevalence is doubled in children with risk factors such as obesity, craniofacial syndromes, Prader-Willi syndrome or trisomy 21. The most common aetiology of OSAHS in children is tonsil hypertrophy, and the first line treatment proposed for the majority of patients is the surgical removal of these tonsils. However, the risk of residual OSAHS after surgery is approximately 10–20%, and, thus, other therapeutic options are being developed to improve patient care. The objective of this scoping review is to assess the extent of the evidence regarding the effectiveness of the different types of treatments offered for OSAHS in children.

**Methods:**

Relevant studies over a 13 year period were identified using three search engines: PubMed, Scopus and Web of Science. The selection of studies was made using previously defined inclusion and exclusion criteria based on a review of the title and abstracts initially, followed by a full reading of the texts. The studies were classified based on their design and following the grades and level of scientific proof defined by the Health High Authority.

**Results:**

Twenty-nine manuscripts were included for synthesis. The first-line treatment proposed for the majority of patients with OSAHS is surgical removal of the tonsils, but the risk of residual OSAHS after surgery remains significant, and other less invasive options, such as orthodontics, are also useful for improving the management of these patients.

**Conclusion:**

OSAHS treatment recommendations should consider orthodontic treatment as a minimally invasive approach with beneficial effects.

## Introduction

### Definition of sleep Apnoea hypopnoea syndrome (OSAHS)

Obstructive sleep apnoea hypopnoea syndrome (OSAHS) is the most severe stage of obstructive sleep-disordered breathing (OSD), after primary snoring, upper airway high resistance syndrome (UARS) and obstructive hypoventilation syndrome. Specifically, OSAHS is caused by partial (hypopnoea) or total (apnoea) obstruction of the upper airways during sleep [1], leading to an interruption in respiratory flow despite persistent respiratory effort. In 2012, the American Academy of Sleep Medicine (AASM) defined apnoea as a reduction in naso-buccal respiratory flow of greater than or equal to 90% and hypopnoea as a reduction of at least 30% in the amplitude of respiratory flow associated with micro-arousals and a fall in oxygen saturation of greater than or equal to 3% [2–4].

Importantly, the main clinical consequences of OSAHS in children are behavioural problems, including hyperactivity, impaired concentration and learning ability, and delayed growth in height and weight. If left untreated, these problems can worsen and lead to cardiovascular and metabolic complications and, ultimately, metabolic syndrome [1, 5, 6]. Overall, OSAHS significantly impairs patients’ quality of life [7, 8].

The diagnosis of OSAHS is made on the basis of a thorough history and clinical examination, supplemented when possible by a ventilatory polygraph or ideally a polysomnography, as recommended in the AASM report. Questionnaires are also available to help screen for OSAHS, although they cannot be used to make a diagnosis [7, 9]. Furthermore, the apnoea hypopnoea index (AHI), which corresponds to the number of respiratory incidents per hour of sleep, is used to define the degree of severity of OSAHS. Although there is no exact consensus on the definition of stages based on the AHI, a score greater than 1 is considered pathological in children [10], a score between 1 and 5 corresponds to mild OSAHS, a score between 5 and 10 corresponds to moderate OSAHS, and a score above 10 corresponds to severe OSAHS [10, 11]. An AHI greater than 5 is generally associated with daytime sleepiness and learning difficulties in children.

### Prevalence and epidemiology of OSAHS

Epidemiological studies of OSAHS in children are still rare, and the clinical picture of OSAHS is more varied in children than in adults. Therefore, it is difficult to determine the precise prevalence of this syndrome in children. Based on the guidelines of the American Academy of Pediatrics (AAP), the French National Authority for Health estimated that OSAHS affects up to 5.7% of children up to the age of 18 [5], and this prevalence is doubled in children with risk factors such as craniostenosis, Crouzon syndrome, Pierre Robin sequence, Down’s or Prader-Willie syndrome, mucopolysaccharidosis or obesity.

### Treatment of paediatric OSAHS

The diversity of clinical presentations of OSAHS in children makes it difficult to reach a consensus regarding best practice for the management of this condition. There are several approaches for treatment, including surgery, continuous ventilation, medication, and orthodontics, as well as myofunctional rehabilitation and specific treatments for obese patients. However, depending on the patient’s age, the severity of the OSAHS and the presence of risk factors, not all options are feasible for every patient. It is important to note that the policy for diagnosis varies from country to country, with many countries requiring a medical doctor to carry out the diagnosis, and how the diagnosis is required prior to any dental or orthodontic intervention.

#### Surgical treatments

The main aetiology of OSAHS in patients with no risk factors is hypertrophy of the tonsils [12, 13], and surgical removal, in particular adenotonsillectomy (removal of the palatal and pharyngeal tonsils), is often proposed as a treatment strategy [13, 14]. Other approaches, such as pharyngeal plasty, reduction of the inferior turbinate, tongue reduction, supraglottoplasty or orthognathic surgery exist, but these options are underdeveloped and mainly performed on syndromic patients [14, 15].

#### Treatment with continuous positive airway pressure (CPAP)

A CPAP device delivers a continuous flow of air into the airways by means of a nasal or naso-buccal mask attached to the face [13, 14]. This airflow maintains sufficient pressure in the airways to prevent them from collapsing. This treatment option is particularly suitable for obese, syndromic patients or those with residual post-surgical OSAHS.

#### Drug treatments

Drug therapy using intranasal corticosteroids or anti-leukotrienes can be used for treating OSAHS [13–15]. These treatments have proved effective in relieving symptoms in patients awaiting surgery or in treating mild OSAHS, but they are generally only short-term options [16–18].

#### Orthodontic treatment

Orthodontic treatment offers an effective therapeutic approach for OSAHS, particularly for patients with dento-maxillary disharmony [19]. These orthodontic treatments include rapid maxillary expansion (RME) appliances, mandibular advancement orthoses (OAM), or a combination of the two [15, 19]. The aim of such treatment is to recover normocclusion in order to clear the oropharyngeal carrefour and prevent posterior tongue thrust [19]. Notably, orthognathic surgery is mainly performed on syndromic patients [14].

#### Specific treatments for the paediatric population with obesity

As obesity is a risk factor for OSAHS, weight loss is often a first step in treatment, as a prelude to another therapeutic option, such as surgery [20, 21]. Furthermore, recent studies of positional sleep therapy in obese children with OSAHS have shown encouraging results [22, 23].

#### Myofunctional rehabilitation

Myofunctional re-education is a complementary therapy for OSAHS that has been developing over recent years. Specifically, this treatment helps to establish physiological nasal breathing rather than mouth breathing. Although myofunctional re-education is not a treatment option on its own, it appears to support the results obtained following surgical or orthodontic treatment, thereby preventing the recurrence of OSAHS [24–28].

**Fig. 1:**
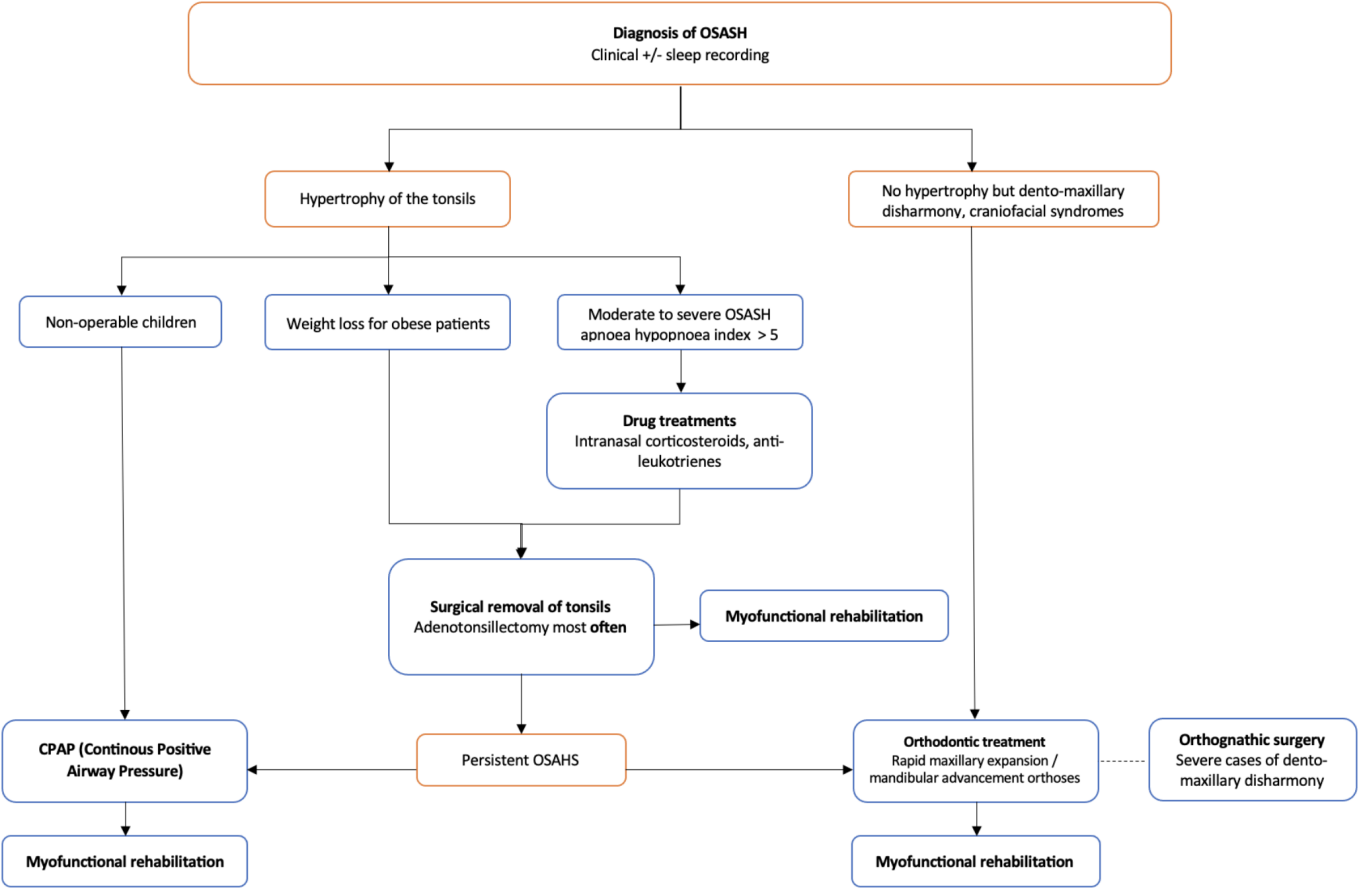
Proposed decision tree for the management of paediatric OSASH (personal iconography)

The proposed decision tree summarises the treatment options available for the management of OSAHS in children (Fig. 1).

### Objective

The current study aimed to summaries the evidence on the effects of treatments on outcomes in OSAHS pediatric patients. The goal was to provide an overview of all types of approaches available in the literature for the management of OSAHS pediatric patients. The question to be answered was: can orthodontics play a role in the management of OSAHS in pediatric patients and what evidence is available regarding its effectiveness?

## Methods

### Design

This review is identified as a scoping review as mainly follow a systematic approach to map evidence on a topic and identify main concepts, theories, sources, and knowledge gaps. The Preferred Reporting Items for Systematic reviews and Meta-Analyses extension for Scoping Reviews (PRISMA-ScR) Checklist were followed in the study (appendix) [29].

### Eligibility criteria

According to the Joanna Briggs Institute (JBI), the population, concept, context (PCC) framework is recommended as a guide to construct a clear and meaningful protocol for a scoping review. The PCC design of the eligibility criteria were as follows:

Population: participants aged < 18 years with a diagnosis of OSAHS (defined by obstructive AHI ≥ 1 event/h).

Concept: at least one therapeutic option investigated with an assessment of the effects of treatment (on AHI), or at least a trend with a level of evidence A or B (RCTs, systematic reviews and meta-analysis, decision analysis based on well-conducted studies, well-conducted comparative studies, cohort studies).

Contexts: OSAHS as the main pathology (excluding syndromes and specific conditions).

### Identification of studies

This work involved searching for keywords using the MeSH database in order to create the search queries. The inclusion criteria were as follows: articles written in French or English over a period extending from 1 January 2011 to 1 December 2024 and containing the words “sleep apnoea”, ‘hypopnea”, “child”, “pediatric” and “treatment”. We explored three databases, including PubMed ((“sleep apnea“[Title/Abstract]) OR (“hypopnea“[Title/Abstract])) AND ((child[Title/Abstract]) OR (pediatric[Title/Abstract])) AND (treatment[Title/Abstract]) AND (treatment[Title/Abstract]), Web of Science ((“sleep apnea” OR hypopnea) AND (child OR pediatric) AND treatment) and Scopus ((“sleep apnea” OR hypopnea) AND (child OR pediatric) AND treatment). In these last two databases we applied the ‘Title’ filter to avoid noise.

### Study selection and data extraction

Two trained authors (MP and TM) independently searched the literature for relevant studies to include in this scoping review. Before starting to examine the articles, MP and TM calibrated themselves on 5 articles. Basis on inclusion criteria, eligibility of studies was performed separately by MP and TM. Title and abstracts were reviewed first and the studies that were potentially relevant were each downloaded in full text form. The Manuscripts were independently reviewed by MP and TM for inclusion. In case of disagreement, an advice was taken from a third author (CD). The search results were uploaded into Rayyan software^®^ to facilitate blind selection.

### Data classification and analysis

Data were extracted from the manuscripts and documented in a table in Excel^®^ and classified according to the type of study (evidence level A and B according to the recommendations of the French National Authority for Health [30]). Characteristics of studies were recorded, including title, author, stidy design, year, therapeutic option, sample size and treatment effectiveness. The main outcome of interest was the change in the AHI. The included articles have been read in full for critical analysis.

## Results

### Presentation of results

**Fig. 2:**
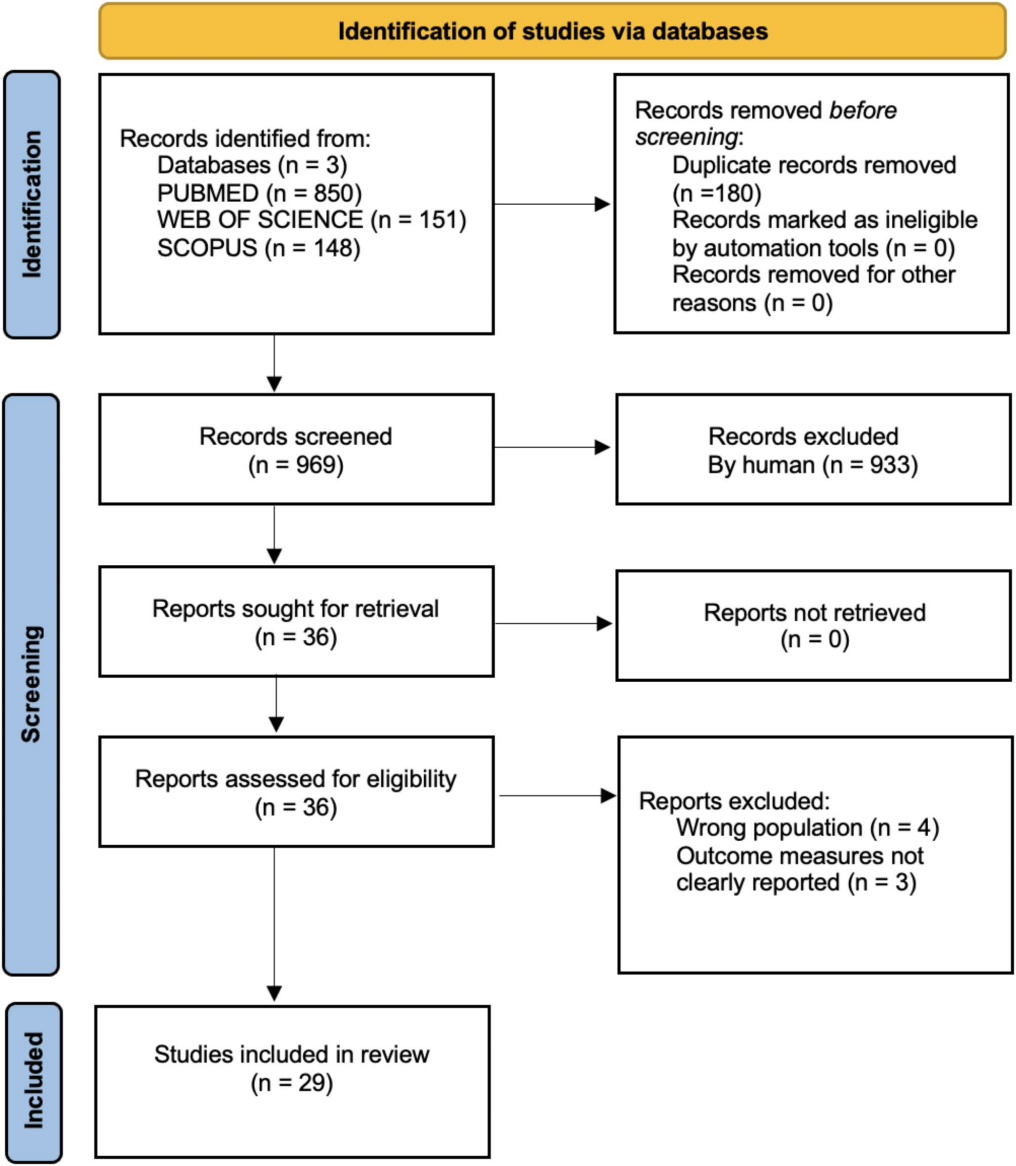
Flow diagram

Figure 2 illustrates the flowchart of the study selection process. A total of 1149 articles were identified, 850 in PubMed, 151 in Web of Science and 148 in Scopus. There were 969 articles remaining after duplications were removed. Irrelevant articles were excluded after reading the title and abstract in Ryyan software^®^, and 36 articles were assessed with full text. Seven articles were excluded due to the wrong population (*n* = 4), and due to outcomes measures not clearly reported (*n* = 3). A total of 29 articles were included in the scoping review. The characteristics of the included studies are presented in Table 1.

**Table 1:** Data analysis of included articles

Article title	Authors	Type of study Reference	Pub. date	Therapeutic option(s)	Sample	Treatment effectiveness
Pharmacological interventions for pediatric obstructive sleep apnea (OSA): Network meta-analysis	Y Zhang, S Lengc, Q Hu, Y Li, Y Wei, Y Lu, D Qiea, F Yang	Network meta-analysis [31]	2024	**Pharmacological intervention** (Mometasone, + Montelukast, Budesonide)	1637	Significant reduction in AHI with Mometasone + Montelukast (-4.74), Budesonide (− 3.45) and Montelukast (-3.41).
Orthodontic appliances for the treatment of pediatric obstructive sleep apnea: A systematic review and network meta-analysis	M Yu, Y Ma, Y Xu, J Bai, Y Lu, F Han, X Gao	Network meta-analysis [33]	2023	**Orthodontics** (rapid maxillary expansion, mandibular advancement device, myofunctional therapy or combined)	595	Reductions in AHI of − 2.18/h with MAA. Significant decrease in AHI (− 5.13/h) with RME + adenotonsillectomy and RME + MAA (-3,79/h). Significant decrease in AHI (− 2.45/h) with MFT. No significant AHI reduction with RME alone.
Management of paediatric obstructive sleep apnoea: A systematic review and network meta-analysis	SY Lin, YX Su, YC Wu, JZC Chang, YK Tu	Network meta-analysis [32]	2019	**Surgery** (Adenotonsillectomy, adenotonsillectomy with pharyngoplasty, adenotonsillotomy), **Pharmacological intervention** (antimicrobial therapy, intranasal corticosteroids, leukotriene receptor antagonists, steroids + LTRAs), **Orthodontics** (rapid maxillary expansion) **Other** (placebo and no treatment)	1064	Surgical removal of hypertrophic adenoids/tonsils is the most effective treatment. Evidence is insufficient to conclude orthodontic intervention as a routine treatment option. RME may not be curative, it is less effective in reducing AHI; however, RME could be helpful in improving breathing and Hypoxaemia.
Mandibular Advancement Appliances in Pediatric Obstructive Sleep Apnea: An Umbrella Review	C Cozzi-Machado, FR Albertini, S Silveira, AJ Machado-Júnior	Umbrella Review [36]	2023	**Orthodontics** (mandibular advancement appliances)	32 to 269	Mandibular advance- ment appliances improve the AHI and increase posterior airway space, reducing airway collapsibility.
Rapid maxillary expansion in pediatric patients with obstructive sleep apnea: an umbrella review	DF Barbosa, L Fernandes Bana, MC Buta Michel, M Meira e Cruz, E Zancanella, AJ Machado Júnior	Umbrella Review [37]	2023	**Orthodontics** (rapid maxillary Expansion)	102 to 1064	RME improve the AHI but is not recommended for treating OSAHS due to a lack of evidence.
Dental Appliances for the Treatment of Obstructive Sleep Apnea in Children: A Systematic Review and Meta-Analysis	D Marciuc, S Morarasu, BC Morarasu, EA Marciuc, BI Dobrovat, V Pintiliciuc-Serban, RM Popescu, FC Bida, V Munteanu, D Haba	Meta-analysis [38]	2023	**Orthodontics** (dental appliances)	280	The AHI was significantly lower in the Dental appliances group with a mean difference of 5.44, (95%)
Effectiveness of functional orthopedic appliances as an alternative treatment among children and adolescents with obstructive sleep apnea: Systematic review and meta-analysis	R Bernardes, LM Di Bisceglie Ferreira, AJ Machado Júnior, MH Jones	Meta-analysis [39]	2023	**Orthodontics** (functional jaw orthopedic appliances)	310	Reduction of AHI of -1,55.
Effect of orthopedic and functional orthodontic treatment in children with obstructive sleep apnea: A systematic review and meta-analysis	R Bucci, R Rongoa, B Zunino, A Michelotti, P Bucci, G Alessandri-Bonetti, S Incerti-Parent, V D’Anto	Meta-analysis [40]	2022	**Orthodontics** (rapid maxillary expansion, mandibular advancement device, myofunctional therapy or combined)	11 to 110	No significant evidence.
High-flow nasal cannula therapy for pediatric obstructive sleep apnea: a systematic review and meta-analysis	F Du, YH Gu, YC He, WF Deng, ZZ Liu.	Meta-analysis [41]	2022	**Other** (heated and humidified high-flow nasal cannula therapy)	67	Statistically significant reduction in AHI with heated and humidified high-flow nasal cannula therapy (MD: 15.58 95%).
The Efficacy of Twin-Block Appliances for the Treatment of Obstructive Sleep Apnea in Children: A Systematic Review and Meta-Analysis	J Duan, W Xia, K Yang, X Li, F Zhang, J Xu, Y Jiang, J Liang, B Li	Meta-analysis [59]	2022	**Orthodontics** (twin-block appliance)	170	Significant decrease in AHI (4.35 events/h) after twin-block therapy.
Effect of myofunctional therapy on children with obstructive sleep apnea: a meta-analysis	A Bandyopadhyaya, K Kaneshiro, M Camacho	Meta-analysis [42]	2020	**Orthodontics** (myofunctional therapy)	241	AHI reduced of 43% from 4.32 (5.2) to 2.48 (4.0) events/hr.
Mandibular advancement appliances for the treatment of obstructive sleep apnea in children: a systematic review and meta-analysis	M Yanyan, Y Min, G Xuemei	Meta-analysis [35]	2019	**Orthodontics** (mandibular advancement device)	34	AHI reduction in the MAA groups for 6 of the 7 studies in the systematic review Mean difference in AHI of -1.75 for the meta-analysis.
Montelukast and Nasal Corticosteroids to Treat Pediatric Obstructive Sleep Apnea: A Systematic Review and Meta-analysis	BJ Liming, M Ryan, D Mack, I Ahmad, M Camacho	Meta-analysis [43]	2018	**Pharmacological intervention** (anti-inflammatory medications: montelukast, intranasal corticosteroids)	668	55% improvement in the AHI with montelukast. 70% improvement in AHI with montelukast and intranasal corticosteroids.
Mandibular advancement for pediatric obstructive sleep apnea: A systematic review and meta-analysis	MW Noller, C Guilleminault, CJ Gouveia, D Mack, CL Neighbors, S Zaghi, M Camacho	Meta-analysis [44]	2018	**Surgery** (mandibular advancement surgeries)	376	AHI surgical cure was seen in 25.5% of patients (and respiratory disturbance index surgical cure was seen in 37.5% of patients).
Lingual tonsillectomy for treatment of obstructive sleep apnea: a meta-analysis	KT Kang, PJ Koltai, CH Lee, MT Lin, WC Hsu	Meta-analysis [45]	2017	**Surgery** (lingual tonsillectomy)	73	17% success rate, 8.9% reduction in AHI.
Rapid maxillary expansion and obstructive sleep apnea: a review and meta analysis	AJ Machado-Junior, E Zancanella, AN Crespo	Meta-analysis [57]	2016	**Orthodontics** (rapid maxillary expansion)	215	Average reduction in AHI after treatment of 6.86.
Treatment outomes of supraglottoplasty for pediatric obstructive sleep apnea: a meta analysis	CF Lee, Wei-C Hsu, HH Lee, MT Lin, KT Kang	Meta-analysis [47]	2016	**Surgery** (supra-glottoplasty)	121	28% AHI < 1, mean reduction in AHI of 8.9.
Rapid Maxillary Expansion for Pediatric Obstructive Sleep Apnea: A Systematic Review and Meta-Analysis	M Camacho, ET Chang, SA Song, J Abdullatif, S Zaghi, P Pirelli, V Certal, C Guilleminault,	Meta-analysis [48]	2016	**Orthodontics** (rapid maxillary expansion)	314	AHI decreased from a mean 6 standard deviation (M 6 SD) of 8.9 +/- 7.0/hr to 2.7 +/- 3.3/hr (70% reduction).
Orthodontics treatments for managing obstructive sleep apnea syndrome in children: A systematic review and meta-analysis	NT Huynh, E Desplats, FR Almeida	Meta-analysis [34]	2016	**Orthodontics** (mandibular advancement and rapid maxillary expansion)	39	Average variation in AHI of 5.11 for the OAM group. In the RME group, heterogeneity of results but decrease in AHI in all studies after treatment.
Combined Surgical and Orthodontic Treatments in Children with OSA: A Systematic Review	L Templier, C Rossi, M Miguez, J De la Cruz Pérez, A Curto, A Albaladejo, M Lagravère Vich	Systematic Review [49]	2020	**Surgery AND Orthodontics** (adenotonsillectomy) and rapid maxillary expansion and mandibular advancement)	80	Higher decrease in the AHI after both treatments (surgery and orthodontic treatment).
Anti-inflammatory medications for the treatment of pediatric obstructive sleep apnea	S Kuhle, DU Hoffmann, S Mitra, MS Urschitz	Systematic review [50]	2020	**Pharmacological intervention** (Montelukast and intranasal corticosteroids)	240	AHI reduction of 3.41 for montelukast with a moderate level of evidence and a reduction of 3.18 for intranasal corticosteroids with a low level of evidence.
Systematic review of site of obstruction identification and non-CPAP treatment options for children with persistent pediatric obstructive sleep apnea	PV Manickam, SR Shott, EF Boss, AP Cohen, JK Meinzen-Derr, RS Amin, SL Ishman	Systematic review [51]	2015	**Surgery AND Pharmacological intervention** (supra-glottoplasty, lingual tonsillectomy, intra-nasal anti-leukotriene and corticosteroids, radiofrequency ablation of the base of the tongue)	960	AHI < 5 in 58 to 72% of patients after supraglottoplasty, AHI < 5 in 57 to 88% after lingual tonsillectomy, resolution of OSAHS in 61% of patients after radiofrequency ablation of the base of the tongue.
Obstructive sleep apnea in obese children and adolescents, treatment methods and outcome of treatment - A systematic review	IG Andersen, JC Holm, P Homøe	Systematic review [52]	2016	**Surgery** (adenotonsillectomy) **Other** (weight loss, continuous positive airway pressure (CPAP))	1220 patients (16 studies)	Decrease in AHI after TA, but higher residual OSAHS in obese children. Weight loss improves the AHI. CPAP improves respiratory parameters but long-term compliance is difficult
Effectiveness of functional orthodontic appliances in obstructive sleep apnea treatment in children: literature review	RCB Bariani, R Bigliazzi, M Cappellette Junior, G Moreira, RR Fujita	Review [53]	2021	**Orthodontics** (functional orthodontic appliances)	281	Non-statistical reduction in AHI in 12 of the 13 studies, but great heterogeneity of results. Only one study (*n* = 1) saw the AHI increase from 2.6 to 10.2.
Adenotonsillotomy versus adenotonsillectomy in pediatric obstructive sleep apnea: A 5-year RCT	I Sjölander, A Borgström, P Nerfeldt, D Friberg	Randomized controlled trial [54]	2022	**Surgery** (adenotonsillotomy versus adenotonsillectomy)	45	After adenotonsillectomy, AHI decreased from mean 12.3(SD 8.0) to 0.6(0.7), and after adenotonsillotomy from 12.6(7.4) to 0.5(0.6).
Systemic corticosteroids could be used as bridge treatment in children with obstructive sleep apnea syndrome waiting for surgery	M Evangéliste, M Barreto, G Di Nardo, M Del Pozzo, P Parisi, VM Pia	Randomised controlled trial [55]	2021	**Pharmacological intervention** (beclomethasone intranasal spray, Beclometasone intranasal spray + oral betamethasone)	28	Improvement in the AHI. Systemic betamethasone associated with intranasal steroids is effective as rescue treatment in children.
Clinical observation of soft palate pharyngoplasty in the treatment of obstructive sleep apnea hypopnea syndrome in children	XX Ding, LQ Zhao, XG Cui, Y Yin, HA Yang	Randomised controlled trial [56]	2020	**Surgery** (adenoidectomy + tonsillectomy + pharyngoplasty vs. tonsillectomy + adenoidectomy)	150	94.7% cure rate in the pharyngoplasty group compared with 90.7% in the control group.
Randomized controlled study of a mandibular advancement appliance for the treatment of obstructive sleep apnea in children: A pilot study	AJ Machado Júnior, LG Signorelli, E Zancanella, AN Crespo	Randomised controlled trial [57]	2016	**Orthodontics** (mandibular advancement device vs. no treatment)	14	Reduction in AHI for 82% of the experimental group after 1 year and AHI < 1 for all these children. 20% increase in AHI in the control group.
Comparison of treatment modalities in syndromic children with Obstructive Sleep Apnea-A randomized cohort study	SS Sudarsan, VK Paramasivan, SV Arumugam, S Murali, M Kameswaran	Prospective randomised cohort study [58]	2014	**Surgery AND Other** (adenotonsillectomy (AT) vs. continuous positive airway pressure)	80	AHI < 1 for 89.04% on average: 91.89% for AT and 86.11% for CPAP.

AAM: mandibular advancement device, AT: adenotonsillectomy, AHI: apnoea hypopnoea index, OAM: mandibular advancement orthosis, CPAP: continuous positive airway pressure, Hr: hour, LTRAs: leukotriene receptor antagonists, MFT: myofunctional therapy, RME: rapid maxillary expansion, SAHOS: sleep apnoea hypopnoea syndrome, SCR: sleep clinical record, SD: standard deviation, SpO_2_: oxygen saturation, Vs: versus

These articles included three network meta-analyses [31–33], two umbrella review [34, 35], three systematic reviews [36, 37], 14 meta-analysis [28, 31, 34, 35, 38–47], four systematic review [48–51], one review [52], four randomised controlled trials [53–56], one randomised cohort study [57]. The orthodontic option was examined in 16 articles [28, 32, 33, 35–39, 41, 45, 47, 48, 52, 56, 58], surgery in 10 articles [32, 43, 44, 46, 48, 50, 51, 53, 55, 57], and pharmacological drugs in 6 articles [21, 31, 42, 49, 50, 54]. Finally, 4 articles discussed other options: weight loss [51], continuous positive airways pressure [51, 57], heated and humidifier high-flow nasal canula therapy [40], placebo and no treatment [32]. The sample size of theses studies varied from a minimum of 11 [28] to a maximum of 1,637 patients [31].

Among the studies, all of them investigated a specific class of treatment option (surgery or orthodontics or medication). Only 5 studies combined different approaches [32, 48, 50, 51, 57]. The most frequent option in this scoping review was the orthodontic treatment (16 out of 29 articles). There were 11 articles on orthopaedic appliances for mandibular advancement [33–36,38–40,49,53,5759], 8 articles on rapid maxillary expansion [28, 32–34, 37, 45, 47, 48], and 3 articles on myofunctional therapy [28, 33, 41]. Regarding the surgical option, 6 articles concerned adenotonsillectomy [32, 48, 51, 53, 55, 57], 2 articles concerned adenotonsillotomy [32, 53], 2 articles concerned lingual adenoidectomy [44, 50], 2 articles concerned pharyngoplasty [32, 55], 2 articles concerned supraglottoplasty [46, 50], one article concerned tongue base surgery [50] and one article concerned mandibular advancement surgery [43]. Finally, regarding the drug treatment option: 6 studies investigated corticosteroids [31, 32, 42, 49, 50, 54], mainly intranasal (Mometasone, Budesonide, Beclomethasone, Betamethasone and other steroids), 4 studies investigated anti-leukotrienes [31, 32, 42, 49] (Montelukast for example) and one investigated anti-microbial treatments [32].

### Analysis of treatment effectiveness

The results of this study were analysed according to the type of treatment examined, and the different therapies were compared in terms of their effectiveness. The main criterion for evaluating the efficacy of an OSAHS treatment was a reduction in the AHI.

Among the studies investigating surgical approaches to treatment, 6 explored adenotonsillectomy [32, 48, 51, 53, 55, 57], with the results converging to suggest a reduction in the AHI scores following treatment. For example, the study by Ding et al. compared the efficacy of adenotonsillectomy combined with pharyngoplasty with adenotonsillectomy alone and reported that OSAHS was cured in 94.7% of patients in the pharyngoplasty group and 90.7% of patients without [55]. Although the results of both approaches were conclusive, the combination of adenotonsillectomy with pharyngoplasty offers better results for the resolution of OSAHS. Moreover, the study by Sudarsan et al. obtained similar results, with an AHI of less than 1 observed in 91.89% of patients undergoing adenotonsillectomy compared with 86.11% of those treated with CPAP [57]. Notably, according to Andersen et al., the mean reduction in AHI scores after adenotonsillectomy was 15.3, but recurrence was observed in 33–76% of patients with obesity compared with 15–37% of non-obese patients [51]. In the same study, the reduction in AHI after CPAP treatment was 22.8, but there were difficulties with adherence over the long term. Some articles focusing on CPAP reported use periods of less than 4 h per night [51, 57].

Two studies also looked at lingual tonsillectomy as a treatment for OSAHS [44, 50]. For example, Manickam et al. obtained a success rate of 57–88%, considering an AHI of less than 5 as an indicator of surgical success [50]. Kang et al., who studied lingual tonsillectomy as a treatment for persistent OSAHS after adenotonsillectomy, obtained a success rate of 17%, with an AHI of less than 1 or 5, depending on the study, as the cure criterion [44]. The study by Manickam et al. also explored supraglottoplasty as a treatment option for OSAHS and obtained a success rate of 58–72% of patients [50]; however, Lee et al. found that only 28% of patients had an AHI of less than 1 after supraglottoplasty [46]. Manickam et al. also studied other surgical approaches, such as radiofrequency ablation of the base of the tongue, which resulted in the resolution of OSAHS in 61% of patients, and subperiosteal release of the lingual floor muscles in patients suffering from the Pierre Robin sequence, resulting in an improvement in respiratory obstruction in 84% of patients [50].

Further to surgical approaches, among the studies of orthodontic treatment, 8 evaluated RME [28, 32–34, 37, 45, 47, 48]. Specifically, Machado-Júnior et al. conducted a meta-analysis of case-control studies on RME and found an average AHI reduction of 6.86 in children treated with a rapid maxillary expansion device [45]. Huynh et al. also analysed the efficacy of RME in the treatment of OSAHS and found a reduction in AHI in all studies, although the results varied widely and do not permit conclusions to be drawn about the effectiveness of this treatment option [32, 34, 37]. This same study also explored the efficacy of mandibular advancement treatment (MAAT) and reported a 5.11 reduction in AHI between the start and end of orthodontic treatment. Several other studies have examined the effectiveness of orthopaedic appliances for mandibular advancement [33–36,38–40,49,53,5759]. For instance, Machado-Júnior et al. reported an 82% reduction in AHI in an experimental group, with an AHI of less than 1 for all patients, while AHI increased by 20% in the control group [56]. A network meta-analysis from 2023 confirms this trend in correcting AHI [33]. If Bandyopadhya et al. observed a reduction in AHI of 43% with myofunctional therapy [41], Other studies, such as the meta-analysis of Bucci et al., do not provide sufficiently strong evidence of its efficacy [28].

As another treatment modality, several studies examined the efficacy of drug treatments, in particular anti-leukotrienes and intranasal corticosteroids, for treating OSAHS. The study by Kuhle et al. compared montelukast (an anti-leukotriene) and intranasal corticosteroids against placebo and obtained a reduction in AHI of 3.41 for montelukast and 3.18 for corticosteroids compared to controls [49]. Additionally, Manickam et al. studied the combined action of anti-leukotrienes and intranasal corticosteroids and observed a 3.6 reduction in AHI [50]. Evangelisti et al. compared the efficacy of intranasal corticosteroids alone and in combination with an oral corticosteroid by administering an intranasal spray of beclomethasone to one group (G1) and an intranasal spray of beclomethasone with oral betamethasone to a second group (G2) [54]. The authors reported a better improvement in outcomes on the Sleep Clinical Record (SCR), which combines a physical examination with the patient’s subjective symptoms and medical history, for the G2 group compared with the G1 group. A more network meta-analysis also highlighted that the reduction in AHI was greater with the combination of corticosteroids and antileukotrienes (-4.74) compared with corticosteroids alone (-3.45) or antileukotrienes alone (-3.41) [59, 60].

## Discussion

The aim of this review was to provide an overview of all types of approaches available in the literature for the management of OSAHS paediatric patients to examine the role of orthodontics more closely.

As for the selection criteria for the articles, the studies were not excluded based on the quantitative nor qualitative aspects of the population samples as long as the populations involved children aged 0 to 18 years old. However, the inclusion ages of the patients differed between studies, making it difficult to generalise the data. The difficulty in recruiting patients and the current underdiagnosis of this condition also resulted in small sample sizes, making large-scale extrapolation of the results unfeasible. Moreover, some articles chose to exclude specific populations of children with certain risk factors, but the same treatment may have different results depending on the population studied, thus making it difficult to reach a consensus on the efficacy of each treatment. The absence of a control group did not lead to the exclusion of articles, since for certain studies, a control group could pose an ethical problem with regard to the potential harm to patients not receiving the treatment.

In terms of diagnosis, the use of preoperative polysomnography, although considered the gold standard for diagnosis, was not a necessary inclusion criterion for studies. It should be noted that if the AHI represents an objective criterion for evaluating the efficacy of a treatment, this index requires polysomnography or ventilatory polygraphy, ideally performed in a sleep laboratory in a hospital or at home. These tests are fairly expensive and difficult to set up, meaning they are not routinely carried out. The cut-off point for the AHI to identify cases cured by treatment was less than 1 or 5 depending on the study, although an AHI greater than 5 is significantly associated with sleepiness and learning difficulties according to Franco et al. [2]. Notably, there is no real consensus on the pathological cut-off point for the AHI in children.

Regarding surgical intervention, adenotonsillectomy is still the most commonly used treatment option and recommended as first-line treatment in case of adenotonsillar hypertrophy [26]. Indeed, adenotonsillectomy offers good results in patients whose main aetiology is hypertrophy of the palatine tonsils, whether or not this is associated with the pharyngeal tonsils and independent of risk factors. However, the potential post-operative complications must be considered, such as bleeding and pain, which can be difficult to manage in some patients. Moreover, the risk of recurrence of hypertrophy is not negligible, and residual OSAHS is frequently observed, with 10–20% of patients requiring a second course of treatment [61, 62]. One study looked at pharyngoplasty in addition to adenotonsillectomy, which could improve post-operative results by limiting complications, but further studies are required to investigate the long-term benefit of this technique [55]. Other surgical approaches exist for treatment, such as removal of the lingual tonsils, which is generally proposed for persistent OSAHS [61]. Supra-glottoplasty, although less invasive, is less effective in reducing the AHI. In a meta-analysis by Lee et al. [46], 28% of children treated with supra-glottoplasty had a post-operative AHI of less than 1. The effectiveness of adenoidectomy with or without tonsillectomy was estimated at 60% on average (with an AHI less than 1 or 5) in the HAS report in 2012 and at 70–80% in the SFORL report [63, 64]. According to this SFORL report, 40–70% of children experience post-operative nausea and vomiting following tonsillectomy, and post-operative pain assessed using a visual analogue scale (VAS) was found to be greater than 65 mm for the first 24 h and around 50 mm up to day 4, corresponding to a high level of pain. Post-operative haemorrhagic complications, whether immediate (within 8 h) or delayed due to the occurrence of pressure sores (8 to 15 days), also occur in 2–6% of patients [64]. Recommendation number 4 (grade A according to the HAS report) of the 2021 SFORL report states that tonsillectomy with adenoidectomy should be the reference treatment for OSAHS in children with adenoidectomy. The HAS also published a decision tree presenting adenotonsillectomy as the first-line treatment for children with OSAHS and tonsillar hypertrophy [63]. However, in 2002 en France, 68,000 tonsillectomies with or without adenoidectomies were performed in children and adolescents, compared with 35,000 in 2010, so the use of this surgery seems to be declining [63]. Currently though, tonsillectomy with or without adenoidectomy, although invasive, remains the most common treatment and the most effective surgical option.

Further to surgery, CPAP is a treatment option frequently used in patients with mild residual OSAHS after surgery or in patients with contraindications to surgery. The continuous flow of air restores good oxygenation during sleep, significantly improving patients’ quality of life. However, given the difficulty of CPAP implementation, this approach requires the patient’s support network to have a good understanding of the treatment and be involved in its use. Furthermore, most patients can only tolerate the mask for a few hours, and long-term compliance is poor. Marcus et al. evaluated the efficacy of and compliance with CPAP in children, and their study showed a reduction in AHI of around 78% after 6 months of use [65]. However, their work also revealed that 35% of patients dropped out before the 6-month protocol control period. Patients who continued to use the device used it for an average of 5.8 h per night, whereas children aged between 2 and 16 years old tend to sleep an average of 8 h per night. The main causes of non-adherence to CPAP are linked to wearing the mask and the lack of a seal on the ventilation system leading to unintentional leaks. The most frequent adverse effects include skin erythema and even bedsores, eye irritation, nasal dryness, digestive bloating and hypo-development of the facial mass, especially in very young children, due to the compression of the mask [66]. To limit these effects, it is sometimes necessary to vary the type of mask to change the support points. Overall, CPAP is an effective technique for treating OSAHS but is not suitable for young patients in the long term.

Drug treatments, in particular anti-leukotrienes and intranasal corticosteroids, appear to be effective in treating mild to moderate OSAHS for short periods [31]. To date, they are mostly prescribed to relieve symptoms while patients are waiting for another treatment option, often surgery. Studies into their long-term use are required to gain a better understanding of the effects of these treatments and their indications. Indeed, a number of undesirable side effects of corticosteroids are already known. These effects are most often dose- and time-dependent, but they appear in 90% of patients as early as 60 days after taking the drugs. The most common side effects include osteoporosis, hypothalamic-pituitary-adrenal axis disruption, diabetes, the onset of glaucoma, psychiatric or cardiovascular disorders and gastrointestinal or dermatological problems, and these medications also predispose patients to infections because of their immunosuppressive and anti-inflammatory effects [17]. These treatments may suffer with issues regarding long-term compliance in young patients, highlighting the need for therapeutic education.

In the last five years, orthodontic therapy has been developing for the management of patients with OSAHS, particularly those without tonsillar hypertrophy [35–39, 41, 58]. Indeed, it has been shown that physiological respiratory function is more difficult to achieve in patients with maxillary endognathy or retromandibulia [59], and the aim of these orthodontic treatments is to clear the oro-pharyngeal carrefour by preventing the posterior fall of the tongue in order to facilitate the passage of air during breathing. To achieve this, RME or mandibular advancement appliances are used. The majority of studies on orthodontic treatment in this context have shown mixed but encouraging results. In 2023, Barbosa pointed out that although RME improves AHI, it cannot be recommended as a treatment for SAOHS due to the lack of evidence [37]. For example, in their systematic review of mandibular advancement orthoses, Yanyan et al. found a reduction in AHI in six of the seven identified studies on mandibular advancement appliances [35], while Bariani et al. found a non-significant reduction in AHI in 12 of the 13 identified studies on functional orthodontic appliances [52]. For Huynh et al., who studied mandibular advancement orthoses (MAOs) and maxillary expansion appliances (MMEs), a reduction in AHI was observed in all studies, but with heterogeneous results in the MME group [30]. Notably, such treatments are of short to medium duration, lasting approximately 6 to 12 months with an active phase and a retention phase [34, 35, 52]. A meta-analysis of Bucci (2022) highlighted that although orthodontic treatments had positive effects, there was no significant evidence to recommend this treatment option on a routine basis [28]. This supports the results of Lin’s (2019) network meta-analysis [32]. These treatments are also less invasive than surgery but require a minimum of cooperation from the patient and good oral hygiene. While there is insufficient evidence to support the exclusive effectiveness of orthodontic treatment in the management of pediatric OSAHS, orthodontics may have a key role to play in the overall treatment as a complement to surgical interventions. Orthodontics can help improve jaw alignment and tongue position, which may optimise the airway and reduce the risk of recurrence after surgery. It can also correct dental and mandibular abnormalities, facilitating more fluid breathing, especially during sleep. So, while surgery remains the primary treatment, orthodontics plays a complementary role in improving quality of life, preventing future complications and promoting optimal development of oro-pharyngeal structures, strengthening the global approach to treating pediatric. Orthodontists, with their deep understanding of malocclusions in the context of the intermaxillary relationship, tongue position, bone morphology and airway, can create customised treatment plans for their patients. To this end, orthodontic treatment using invisible aligners has developed considerably in recent years, but there is currently no evidence of their therapeutic effects in OSAHS. A retrospective study published in 2022 nevertheless shows encouraging results with this type of treatment [67]. Therefore, further studies are needed to better understand the outcomes of these orthodontic appliances, particularly in syndromic patients or those with obesity, as well as their long-term results.

A few articles have studied weight loss as a treatment for patients with obesity, including behavioural ( (i.e. dietary restriction, physical activity and psychological support) [68, 69], and surgical (i.e. sleeve, bariatric surgery) interventions [22, 70], and these treatments have always resulted in an improvement in AHI. However, further studies are needed to refine the parameters that lead to an improvement in symptoms in these patients. Other new therapeutic approaches also continue to emerge, in particular concerning orofacial myofunctional re-education in parallel with other therapies to improve the AHI and clinical symptoms of patients [71–73]. Indeed, poor lingual positioning is often the cause of atypical or infantile swallowing, leading to a spiral of dysmorphic dysfunction. As a result, restoring a physiological lingual position can maintain or improve the results obtained using orthodontic or surgical treatment. Additionally, postural re-education with a physiotherapist and exercises designed to improve the tone of the orofacial muscles can be of significant benefit to patients, provided that the exercises are properly followed. Overall, these studies demonstrate the need for healthcare professionals to work as part of a multidisciplinary team to provide the best possible care for children suffering from OSAHS [8].

Finally, new examination techniques have been developed to refine the diagnosis of OSAHS, such as sleep endoscopy (DISE) [50, 52, 62]. This technique is a pharyngolaryngeal fibroscopy performed under general anaesthetic, and it provides better identification of the site of airway obstruction in sleep conditions. Another alternative is cine-MRI, which provides a dynamic analysis of the airways in three dimensions under induced sleep [50]. These new diagnostic approaches could clarify the type of therapeutic approach to be adopted and avoids ineffective treatments, particularly surgery in some contexts.

The research yielded a limited number of results, considering the diversity of possible treatments and the period over which the articles were included (13 years). This can be explained by the level of evidence of the articles included (grade A and B), thus excluding numerous cross-sectional studies and case reports. One of the main limitations of scoping reviews is that they often include studies using different methodologies (observations, clinical trials, case reports, etc.). The review may therefore include research of varying quality, which may influence the strength of the conclusions. However, despite these limitations, the scoping review remains a valuable tool for mapping a field of research, identifying gaps in knowledge, and guiding the development of future research. It guides researchers and clinicians towards important research questions, even if it requires caution in terms of recommendations.

## Conclusion

The management of OSAHS in children represents a major therapeutic challenge because of the numerous repercussions for patients’ quality of life and health. OSAHS in children also predisposes them to OSAHS in adulthood and to the development of cardio-respiratory pathologies. The difficulty of managing OSAHS in children stems from the variable clinical picture and the many risk factors involved in its development. Early detection would enable better management of the most severe cases while limiting the secondary effects, particularly in terms of growth restrictions. Surgery is still often the first-line treatment for tonsillar hypertrophy, but the development of other less invasive treatment options, particularly dentofacial orthopaedics, is improving the management of patients with OSAHS.

The results of this scoping review provide a global vision for clinicians treating OSAHS. By considering various treatments (surgical, orthodontic and pharmacological), this study highlights the need for a personalised assessment of patients’ needs. A proper diagnosis by a medical doctor would help determine the proper path of treatment. This paper provides alternative solutions (such as orthodontic appliances) that may be less invasive than surgery. In addition, the integration of these complementary approaches would promote more stable long-term results, reducing the risk of recurrence of symptoms and improving children’s quality of life. This review therefore supports the need for multidisciplinary and personalised management, which is essential for optimising the treatment of OSAHS. It supports the need to develop further studies based on orthodontic treatment in order to obtain evidence-based recommendations.

## Data Availability

The bibliographic data supporting this research are cited in the references (including DOIs when they exist).
